# Cross-Serogroup Analysis of Representative Top-Seven Shiga toxin-producing Escherichia coli Plasmids Reveals Lineage-Specific Patterns

**DOI:** 10.64898/2026.09.16.752139

**Published:** 2026-09-18

**Authors:** Ali Nemati, Irvin Rivera, Soren Mohammadi, Ali Dadvar, Nima Dehdilani, Sara S. K. Koenig, Adeleh Arzhangi, Anwar Kalalah, Ali Sardarzadeh Majd, Amiryazdan Taghizadeh Shool, Ute Römling, Federica Gigliucci, Mark Eppinger

## Abstract

Plasmids play a significant role in shaping the pathogenic potential of Shiga toxin-producing Escherichia coli (STEC) by encoding virulence and adaptive genes that complement chromosomal determinants. While plasmid-encoded factors such as ehxA, espP, katP, and toxB are known to enhance intestinal colonization and host interaction, the diversity and evolutionary patterns of STEC plasmids remain insufficiently characterized. Most previous studies have focused on single serogroups, particularly O157, leaving cross-serogroup comparisons largely unexplored. To address this gap, we conducted an in-depth investigation of 109 complete plasmid sequences (1.5-187 kb) from the "Top Seven" STEC serogroups (O26, O45, O103, O111, O121, O145, and O157), retrieved from NCBI, to examine their structural organization, virulence composition, resistance patterns, mobility potential, and evolutionary dynamics. Our analysis revealed the dominance of F-type plasmids carrying IncFIB and IncFII replicons across serogroups, along with lineage-specific associations with major virulence genes. By examining the enterohemolysin operon across ehxA-positive plasmids, we identified a highly conserved structural framework maintained across 92.5% of analyzed sequences, with minimal variation. We further characterized antimicrobial resistance gene distribution, finding that only 11% of plasmids (12/109) carried such genes, of which 92% were multidrug-resistant. Analysis of mobility features revealed that predicted conjugative potential and MOBF-type relaxases varied considerably among serogroups. The 32 plasmids without virulence or resistance cargo carried at most colicin determinants, yet all received a predicted mobility class, with conjugative machinery confined to plasmids above 37 kb. At the plasmid level, serogroups O26/O103 are closely related and carry relatively high-risk virulence profiles, while the O121/O145 group exhibits moderate virulence gene inventories. The O45/O111 group is further distinguished by its transfer features, while O157 strains form a distinct clade with the broadest array of plasmid-encoded high-risk virulence genes.

## Full Text Availability

The license terms selected by the author(s) for this preprint version do not permit archiving in PMC. The full text is available from the preprint server.

